# The impact of an oral nutritional supplement on body weight gain in older adults with malnutrition: an open-label randomized controlled trial

**DOI:** 10.1186/s13063-023-07622-4

**Published:** 2023-10-02

**Authors:** Ranil Jayawardena, Prasani Wickramawardhane, Chamila Dalpatadu, Andrew P. Hills, Priyanga Ranasinghe

**Affiliations:** 1 Department of Physiology, Faculty of Medicine, University of Colombo, Colombo, Sri Lanka; 2 Health and Wellness Unit, Faculty of Medicine, University of Colombo, Colombo, Sri Lanka; 3 School of Health Sciences, College of Health and Medicine, University of Tasmania, Launceston, Tasmania Australia; 4 Department of Pharmacology, Faculty of Medicine, University of Colombo, Colombo, Sri Lanka; 5 University/British Heart Foundation Centre for Cardiovascular Science, The University of Edinburgh, Edinburgh, UK

**Keywords:** Body weight, Malnutrition, Older adults, Oral nutritional supplementation (ONS), Weight gain

## Abstract

**Background:**

The global aging population is expanding rapidly and many individuals have a particularly higher risk of malnutrition. Malnutrition can lead to impaired body function, morbidity, and mortality. Meeting nutritional requirements is a key strategy to minimize multiple debilitating adverse outcomes associated with malnutrition in the elderly.

Oral nutritional supplements (ONS) have been widely used as a dietary intervention for malnutrition in older adults. These supplements provide additional nutrients and calories to support nutritional requirements and have been shown to improve nutritional status, physical function, and quality of life in malnourished older adults.

**Methods:**

This is an open-label, randomized controlled, parallel-group study including 50 institutionalized older adults (aged > 60 years) with malnutrition or at risk of malnutrition, living in a selected elderly care institution in Colombo, Sri Lanka. The aim is to assess improvement in healthy body weight gain and body composition in older adults with malnutrition at risk of malnutrition by using an ONS. Older adults will be screened for malnutrition using the Mini Nutrition Assessment (MNA) tool and eligible participants randomized using the simple random sampling technique to intervention and control groups (1:1 allocation ratio). The intervention group will consume 200 mL of ONS before bed continuously for 12 weeks. The primary outcome is the percentage who achieved at least 5% weight gain in the intervention group compared to the control group. Nutritional status (anthropometric, biochemical, clinical, and dietary), body composition (dual-energy X-ray absorptiometry), frailty, functional capacity (hand grip strength, knee extension, and Barthel index) cognitive status (Montreal Cognitive Assessment), and physical activity will be assessed as secondary outcomes at baseline and at the end of the 12-week intervention. Some measurements (anthropometry, dietary, and functional assessments) will also be performed at the end of the 4th week. Data will be analyzed using SPSS V-23.

**Discussion:**

This study will determine whether the use of an ONS is effective in promoting healthy weight gain in older adults with malnutrition or at risk of malnutrition. In addition, investigating the impact of an ONS on multiple outcomes via clinical, nutritional, functional, and cognitive function will provide a more comprehensive understanding of the potential benefits of these supplements.

**Trial registration:**

Sri Lanka Clinical Trail Registry SLCTR/2022/021. Oct. 6, 2022.

**Supplementary Information:**

The online version contains supplementary material available at 10.1186/s13063-023-07622-4.

## Administrative information

Note: the numbers in curly brackets in this protocol refer to SPIRIT checklist item numbers. The order of the items has been modified to group similar items (see http://www.equator-network.org/reporting-guidelines/spirit-2013-statement-defining-standard-protocol-items-for-clinical-trials/).

**Table:** 

Title	The impact of an oral nutritional supplement on body weight gain in older adults with malnutrition: an open-label randomized controlled trial
Trial registration	Ethical clearance has been obtained from the Ethics Review Committee of the Sri Lanka Medical Association (ERC/22-005). The trial is registered at the Sri Lanka Clinical Trials Registry (SLCTR/2022/021) Universal Trial Number (UTN) U1111-1282-4903
Protocol version	Protocol version 05 (20/03/2023)
Funding	Financial support for data collection, laboratory investigations from a third party, and ONS will be provided by Kalbe Lanka Pvt. Ltd.
Authors’ details	1. Prof. Ranil Jayawardena – Corresponding author E-mail: ranil@physiol.cmb.ac.lk Professor in Nutrition, Department of Physiology, Faculty of Medicine, University of Colombo, Colombo, Sri Lanka. 2. Ms. Prasani Wickramawardhane Research Assistant, Health and Wellness Unit, Faculty of Medicine, University of Colombo, Colombo, Sri Lanka. 3. Dr. Chamila Dalpatadu Consultant general physician (geriatric) and senior lecturer in Department of Physiology, Faculty of Medicine, University of Colombo, Colombo, Sri Lanka. 4. Prof. Andrew P Hills Professor of Exercise and Sports Science, School of Health Sciences, College of Health and Medicine, University of Tasmania, Launceston, Tasmania, Australia. 5. Prof. Priyanga Ranasinghe Professor in Pharmacology, Department of Pharmacology Faculty of Medicine, University of Colombo, Colombo, Sri Lanka, and British Heart Foundation Centre for Cardiovascular Science, The University of Edinburgh, Edinburgh, UK.
Name and contact information for the trial sponsor	Kalbe Lanka Pvt. Ltd. No. 321, Galle Road, Colombo 4, Sri Lanka. Email: info@kalbesrilanka.com Tel: +94114515614
Role of sponsor	The study sponsor does not have authority over study design; data collection, management, analysis, and interpretation of data; writing of the report; and the decision to submit the report for publication. The study sponsor will provide funding for the conduct of the study and ONS (Entrasol Platinum) formulation.

## Introduction

### Background and rationale

Over the last three decades, the proportion of older adults aged 65 years and above globally increased by nearly 3% [1]. This proportion is projected to rise further from 9.1% in 2019 to 16.7% in 2050, reaching an estimated 1.5 billion older adults [1]. This aging trend will affect Asia the most, with nearly 1.0 billion older adults estimated to be living in Asia by 2050. According to a recent meta-analysis of 240 studies, the pooled prevalence of older adults at risk of malnutrition was 26.5% in the community and 45.6% in healthcare settings globally. The numbers are even higher in Asia, where the prevalence of malnutrition among elders has reached 29.8% in the community, and 48.0% in healthcare settings, respectively [2]. Social isolation, depression, staff control over feeding, use of anorexigenic drugs, and depression cause weight loss in institutionalized older adults [3]. Malnutrition in the elderly is commonly due to one or more of the following factors: inadequate food intake; food choices that lead to dietary inadequacies; illnesses that cause raised nutrient demand, increased nutrient loss, poor nutrient absorption, or a combination of these factors [3]. Nutritional inadequacy in older adults can be caused by a variety of physiological, pathological, sociological, and psychological causes [4].

Physiological changes in the digestive system, oral cavity, lean mass, and basal metabolic rate occur in people as they age [5, 6]. Disorders of the gastrointestinal system ranging from problems with dentition and swallowing to dyspepsia, esophageal reflux, delayed gastric emptying, atrophic gastritis, declined gastric acid secretion, constipation, and diarrhea are associated with poor dietary intake and nutrition malabsorption in the elderly [3, 7]. Loss of lean mass and a decline in basal metabolic rate (BMR) cause poor nutrient utilization and may influence appetite and food intake [3, 8]. Many diseases (e.g., thyroid, cardiovascular, and pulmonary disease) lead to unintentional weight loss by increasing the metabolic demand and reducing appetite and caloric intake [9]. Chronic diseases such as hypertension, diabetes mellitus, and cardiovascular diseases are managed with dietary restrictions and treated with medication that influences food intake [3]. Reduced functional status, both physical and cognitive, affects older adults' ability to shop for food and meal preparation [3], and inadequate social support and social isolation lead to apathy about food. Psychological disorders like depression are well-known causes of anorexia and weight loss [3, 10], and malnutrition resulting from these causes can have severe consequences on the overall health and well-being of the elderly.

Protein-energy malnutrition (PEM) is the most common form of malnutrition in older adults followed by micronutrient deficiencies [11]. PEM-related consequences increase the risk for loss of muscle and bone mass, in turn increasing the possibility of fractures, limitations in mobility, disabilities that ultimately lower capacity for independent living, and increased mortality [12]. Moreover, malnourished older adults are prone to infectious diseases, impaired wound healing, and slowed post-surgical recovery [13]. Hence, early detection of malnutrition and implementation of appropriate interventions are key strategies to reduce morbidity and mortality while improving overall health and quality of life.

Nutritional intervention for the elderly includes dietary counseling, technical or human assistance at mealtime, food fortification, texture adaptation, and prescriptions of energy- and protein-dense oral nutritional supplements (ONS) [14]. According to the guidelines by the European Society for Parenteral and Enteral Nutrition (ESPEN), enteral nutrition (EN) should be the first choice for older adults with normal gastrointestinal function who are malnourished or at risk of malnutrition [15]. As the first choice for EN, a specialized ONS can conveniently enhance the macro- and micro-nutrient intake [16]. A recent meta-analysis by Mengqi Li et al. found that ONS has beneficial effects on the overall appetite, energy intake, and body weight of malnourished older adults [17]. In addition, in a randomized, placebo-controlled trial of older adults in Singapore, a significant improvement in mid-upper arm circumference (MUAC) was observed in the intervention group who received a specialized ONS in comparison to the placebo group [18]. Another study among malnourished older adults with cardiovascular and pulmonary events found that ONS improved hand grip strength and contributed to overall recovery [19]. Most importantly, a study by Yue Zhong et al. showed that ONS cost-effectively extended the lives of malnourished hospitalized patients [20].

In this context, we designed an intervention study to evaluate the impact of ONS on healthy body weight gain (increase in muscle mass and fat mass), along with secondary outcomes in institutionalized older adults in Sri Lanka. In addition to weight gain, this study will assess the impact of an ONS on nutritional, functional, clinical, and cognitive outcomes and provide a comprehensive understanding of the potential benefits of using ONS as a nutrition intervention for malnourished older adults.

## Objectives

### Hypothesis

Providing an ONS for 12 weeks will increase the body weight of institutionalized older adults with malnutrition or at risk of malnutrition at least by 5%.

### General objective

To evaluate the impact of an ONS on the nutritional, clinical, cognitive, and functional status of institutionalized older adults during a 12-week intervention.

### Specific objectives

To investigate the impact of ONS on the following parameters among institutionalized older adults:

i. Anthropometric measurements — height, weight, mid-upper arm circumference (MUAC), calf circumference (CC), waist circumference (WC), and skin fold thickness
ii. Body composition parameters — whole body dual-energy X-ray absorptiometry (DXA) scanning
iii. Biochemical parameters — full blood count (FBC), Hs-CRP (C-reactive protein), total 25 hydroxy vitamin D, serum albumin, and total cholesterol
iv. Functional capacity — hand grip strength, knee extension, and Barthel index score
v. Frailty five-fried model indicator score
vi. Peak expiratory flow rate
vii. Systolic and diastolic blood pressure
viii. Dietary intake — 24-hour dietary recall
ix. Physical activity level — Physical Activity Scale for the Elderly (PASE) and International Physical Activity Questionnaire (IPAQ)
x. Cognitive status — Montreal Cognitive Assessment score (MoCA)

## Trial design

**Fig. 1:**
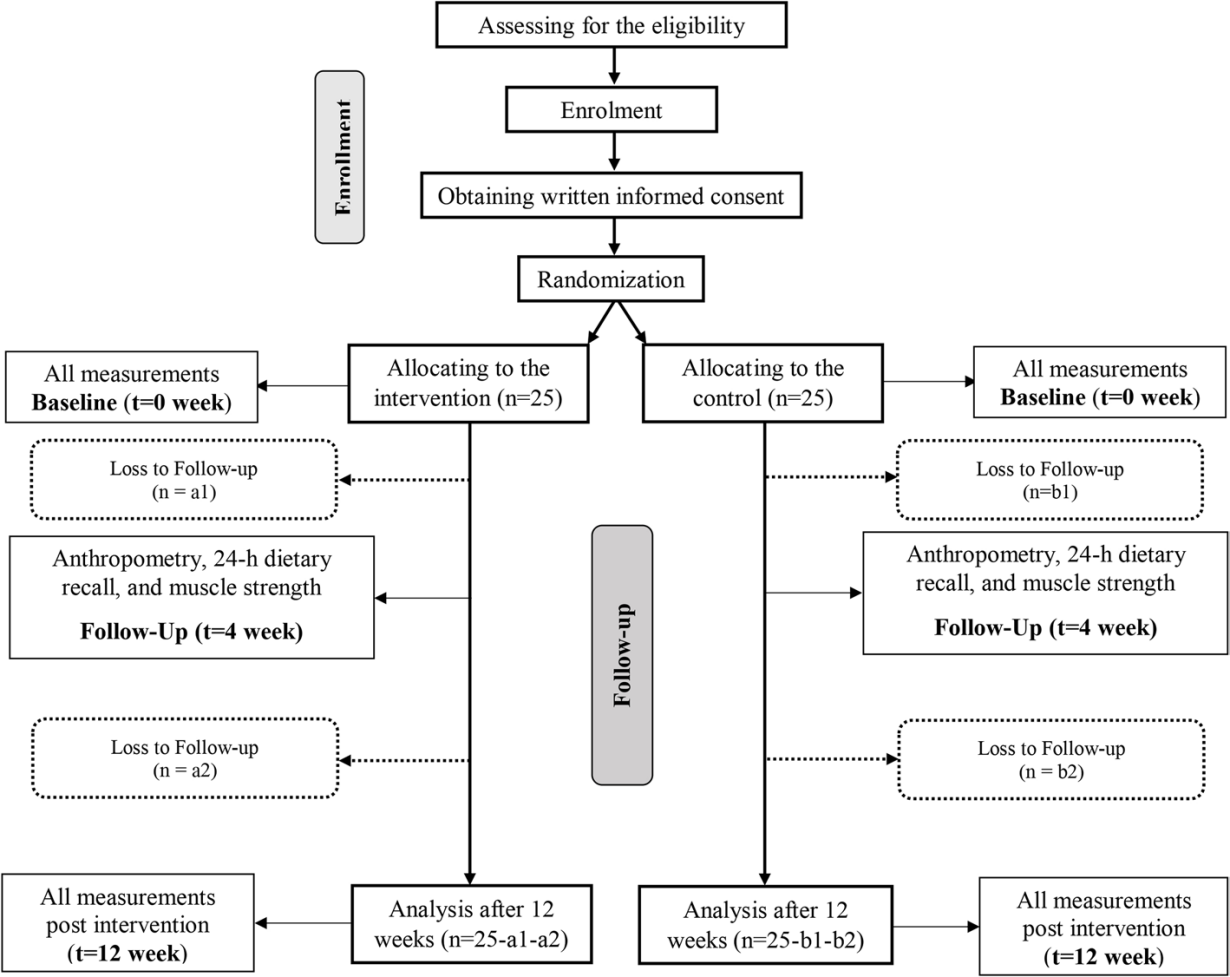
CONSORT flow diagram

This study is a randomized-controlled, parallel-group, clinical trial that will include an intervention group (200 mL of high-energy and protein oral nutrition supplement daily before bed for 12 weeks) and a control group of older adults aged above 60 years who are malnourished or at risk of malnutrition. Participants satisfying the eligibility criteria will be allocated to control and intervention groups equally (1:1). The intervention length is 12 weeks. The CONSORT flow diagram of the trial is provided in Fig. 1. Items included in this protocol conforms to standards described in the SPIRIT (Standard Protocol Items: Recommendations for Interventional Trials) Checklist (Additional File 1).

## Methods: participants, intervention and outcomes

### Study setting

The proposed study will be conducted as an open-label, randomized controlled clinical trial, at a selected residential care facility registered in the National Secretariat for Elders in Colombo, Sri Lanka. Two parallel study groups will include a treatment group and a control group in the same study setting. The study will start with a screening visit to check eligibility.

### Eligibility criteria

Inclusion criteria:

- Aged above 60 years
- Residing in the selected elder’s homes for more than one year
- Malnourished or at risk of malnutrition (MNA score ≤ 11)

Exclusion criteria:

- Presence of chronic illnesses — cardiac failure, stroke, chronic liver disease, and end-stage renal failure or undergoing dialysis
- Those who are unable to respond to the questionnaire due to communication difficulties (elders with speech, hearing, and visual impairment)
- Bedridden
- Who are on a special diet (supplements/tube feed)
- On an end-of-life care pathway
- Without the capacity to consent

### Who will take informed consent?

The investigators (PW, RJ) will obtain informed written consent from participants after explaining the potential benefits and risks of participation and the right to withdraw from the study.

### Additional consent provisions for collection and use of participant data and biological specimens

The information leaflet and consent form include details of the blood samples taken and the tests carried out. No additional consent will be obtained from the participants since biological specimens are not going to be stored for use in future studies.

## Intervention

### Explanation for the choices of comparators

Oral nutritional supplements have been shown to be well tolerated and have a high compliance rate in older adults [21, 22]. Therefore, choosing a specialized ONS can be a convenient and effective way to enhance nutrient intake in this population.

Eligible participants are randomized either into intervention or control group. Participants in the intervention group will consume the ONS before bed daily for 12 weeks in addition to their normal diet. Participants in the control group will be waitlisted and a glass of water will be received before bedtime.

### Intervention description

Before the baseline data collection, both the treatment and control groups will be given dietary advice, during which they will be educated on the importance of including each food group, developing healthy habits, avoiding alcohol and smoking, and maintaining a healthy level of physical activity. Subsequently, they will be randomized to treatment and control groups. During the 12-week study period, participants randomized to the ONS treatment group will receive a 200mL serving of an oral nutrition supplement (prepared by adding 4 level scoops of Entrasol Platinum, Kalbe (Pvt). Ltd (57g) into 200 mL of lukewarm water) daily before bed which contains 12 g protein and 247 kcal per serving, in addition to their usual diet for 12 weeks. The nutritional composition of the ONS is summarized in Table 1.

**Table 1:** Nutritional composition (serving size: 4 scoops [57 g] of ONS)

		**% DV**	**Per serving**	**Per 100 g**
**Total calories**	kcal		247	434
**Total fat**	g	12	8	14
*SFA*	g	23	4.5	8
*MUFA*	g		2	3
*PUFA*	g		1	2
*Trans fatty acids*	g		< 0.5	< 0.5
*Cholesterol*	mg		< 0.5	< 0.5
**Protein**	g	20	12	21
**Total carbohydrate**	g	10	32	55
*Dietary fiber*	g	17	5	9
*Sucrose*	g		7	12.5
*Sodium*	mg	10	153	268
*Potassium*	mg	2.5	126	221
*Vitamin A*	mcg	30	184	323
*Vitamin B*_*1*_	mg	25	0.3	0.55
*Vitamin B*_*2*_	mg	25	0.4	0.71
*Vitamin B*_*3*_	mg	30	4.7	8.2
*Vitamin B*_*5*_	mg	40	2.1	3.6
*Vitamin B*_*6*_	mg	35	0.4	0.75
*Vitamin B*_*7*_*/Biotin*	mg	60	17.6	30.7
*Folic acid*	mcg	30	125	220
*Vitamin B*_*12*_	mcg	30	0.7	1.3
*Vitamin C*	mg	40	26.8	64.5
*Vitamin D*_*3*_	mcg	20	2.8	4.9
*Vitamin E*	mg	30	4.6	8.1
*Calcium*	mg	20	219	384
*Magnesium*	mg	8	26.6	46.6
*Phosphorus*	mg	15	88.7	156
*Iron*	mg	9	2.1	2.7
*Iodine*	mcg	30	46.2	81
*Zinc*	mg	30	3.9	6.8
*Chromium*	mcg	15	3.3	5.8
*Selenium*	mcg	15	4.1	7.2

*SFA* Saturated fatty acids, *MUFA* Monounsaturated fatty acids, *PUFA* Polyunsaturated fatty acids

### Criteria for discontinuing or modifying allocated intervention

A mechanism will be in place to ensure direct reporting of adverse events to investigators through the management of the elder’s home. Participants will be instructed to seek attention immediately. If there is an adverse event related to the study such as nausea, vomiting, diarrhea, bloating, and feeling of fullness, it will be notified immediately to the safety monitoring team, and the patient will exit the study and receive appropriate medical attention. Participants are allowed to withdraw from the study for any reason at any time.

### Strategies to improve adherence to intervention

After starting the intervention, online video calls (WhatsApp or Viber) will be made to caregivers of the selected elderly care institution on a daily basis. The first call will be made to remind participants to ingest either treatment (200 mL of Entrasol Platinum) or control (200 mL of water), after dinner. A second call will be made to make sure whether all participants in the intervention group have consumed the supplement and all the participants in the control group have consumed an equal quantity of water. There will be random visits from the data collection team during the administration of ONS.

Compliance with ONS intake will be calculated over 12 weeks (84 days) using the participant's intake record and return of balance product containers. The percentage of compliance will be calculated by the following formula:

The number of supplement containers consumed is divided by the number of supplement containers instructed to consume, multiplied by 100.

### Relevant concomitant care permitted or prohibited during the trial

Concomitant care apart from the consumption of ONS in the intervention group will be routine care during monthly clinic visits, which includes medication review. Both participants in the intervention and control groups will follow their usual dietary behaviors. Neither their habitual dietary intake nor lifestyle will be altered by the intervention.

### Provisions for post-trial care

We do not expect any adverse effects since the ONS (Entrasol Platinum) used in this study is a locally and internationally marketed product, with no adverse effects reported in post-market surveys. After the intervention period, participants will be screened for malnutrition. If the intervention group derives a benefit by improving nutritional status, the control group will be provided with the ONS after the 12 weeks of the study for 3 months. If the malnutrition status of participants does not improve or worsens, participants will be referred for specialist consultation and management.

### Outcomes

The primary outcome measure is the percentage of intervention group participants who achieved at least 5% weight gain compared to the control group.

The secondary outcome measures include mean between-group differences of the following variables from baseline to the end of the 12th week:

I. Anthropometric measurements
  Body weight
  Height
  Body mass index (BMI)
  Mid-upper arm circumference (MUAC)
  Calf circumference (CC)
  Waist circumference
  Triceps skin fold thickness
II. Body composition
  DXA lean body mass and fat mass
III. Biochemical parameters
  Full Blood Count (FBC)
  Hs-CRP (C-reactive protein)
  Serum albumin
  Total cholesterol
  Serum total 25-hydroxy vitamin D
IV. Functional capacity
  Hand grip strength
  Knee extension
  Activities of daily living — Barthel index
V. Frailty
  Five fried model indicators
VI. Respiratory muscle strength (peak expiratory flow rate)
VII. Energy and macronutrient intake per day
VIII. Cognitive status (Montreal Cognitive Assessment)
IX. Physical activity level
  Physical Activity Scale for the Elderly (PASE)
  International Physical Activity Questionnaire (IPAQ – short form)

### Participant timeline

At baseline and the end of the 12-week intervention period, dietary (24-h dietary recall), anthropometric (weight, body mass index (BMI), MUAC, calf circumference, waist circumference), biochemical (full blood count, serum C-reactive protein, serum albumin, total cholesterol, and serum total 25-hydroxy vitamin D), functional (hand grip strength, knee extension, and Barthel index), frailty (five fried model indicator score), cognitive (Montreal Cognitive Assessment Score), physical activity (Physical Activity Scale for the Elderly and International Physical Activity Questionnaire), body composition (skin fold thickness and dual-energy X-ray absorptiometry scan), and respiratory muscle strength data will be collected. Participants will be followed up at the end of the 4th week of the intervention only for dietary (24-h dietary recall), anthropometric (weight, body mass index (BMI), MUAC, calf circumference, waist circumference), functional (hand grip strength. knee extension) and body composition (skin fold thickness) outcomes. Details of the study schedule are given in Table 2.

**Table 2:** Schedule of enrolment, interventions and assessments

**Timepoint**	**Study period**		
	**Enrolment baseline** ***T***_***0***_	**Intervention period (Time in weeks)**	
		***4w*** ***Follow-up***	***12w***
**Enrollment**			
Mini Nutrition Assessment	X		X
Eligibility screen	X		
Informed consent	X		
Allocation	X		
**Study arms**			
Intervention group	X	X	X
Control group	X	X	X
**Assessments**			
*Sociodemographic characteristics*	X		
*Body weight*	X	X	X
*Height*	X		
*Mid-upper arm circumference*	X	X	X
*Calf circumference*	X	X	X
*Waist circumference*	X	X	X
*Triceps skin fold thickness*	X	X	X
*Dual-energy X-ray absorptiometry (DXA)*	X		X
*Blood parameters (FBC, serum albumin, total cholesterol, Hs- CRP, total 25-hydroxy vitamin D)*	X		X
*Hand grip strength*	X	X	X
*Knee extension*	X	X	X
*Activities of daily living (Barthel index)*	X		X
*Five fried model indicators*	X		X
*Respiratory muscle strength*	X		X
*Blood pressure*	X		X
*Dietary assessment (24-h dietary recall)*	X	X	X
*Cognitive assessment (MoCA)*	X		X
*Physical Activity Scale for the Elderly (PASE) score*	X		X
*International Physical Activity Questionnaire (IPAQ) score*	X		X

*MoCA* Montreal Cognitive Assessment, *FBC* Full blood count, *CRP* C-reactive protein

### Sample size

The sample size calculation was based on studies indicating that a weight gain of 5% can be expected with an ONS intervention, and is sufficient to achieve an overall improvement in nutritional status [23]. A clinically relevant difference was defined as an increase in this fraction to 75% or greater in the treatment arm. Hence, a total of 50 adults will be recruited for the study (alpha, 5%; beta, 10%; drop-out rate, 20%) after the screening for eligibility against inclusion/exclusion criteria. Then, participants will be randomly assigned into two groups (*n*=25 intervention and *n*=25 control). The formula used for sample size calculation was as follows:

$${n}_{B} =\left( \frac{{p}_{A \left( 1- {p}_{A}\right)}}{k} \right)+{\left(\frac{z1- \frac{\propto }{2 } + z1- \beta }{{p}_{A-{P}_{B}}}\right)}^{2}$$

${n}_{B}$ = Sample size in one arm

*P*_*A*_ = Proportion of participants expected to achieve 5% improvement in body weight in the control group (0.3) [23]

*P*_*B*_ = Proportion of participants expected to achieve 5% improvement in body weight in the intervention group (0.75) [23]

*Κ* = Sampling ratio (1:1)

*Z*_*α*_ = Critical value of the normal distribution at *α* (*α* is 0.05 and the critical value is 1.96)

*Z*_*β*_ =Critical value of the normal distribution at *β* (*β* is 0.1)

### Recruitment

Older adults residing in a selected elderly care institution will be screened using the Mini Nutrition Assessment tool (MNA). Older adults who have been identified with a MNA score equal to or less than 11 and satisfied all the other eligibility criteria mentioned above, will be informed about the study both verbally and in writing, in their native language. Information provided will include the aims of the study, participants’ responsibilities, follow-up visits and procedures, potential individual and societal benefits, risks involved, and the ability to withdraw from the study at any time, without any consequences. In addition, opportunities will be provided during this process for any clarification required by the study participants to ensure maximal understanding. Interested participants will be recruited voluntarily. After the written informed consent has been obtained by the co-investigator (PW), participants will be randomly assigned to receive either the oral nutrition supplement or the placebo for 12 weeks.

## Assignment of intervention: allocation

### Sequence generation

Eligible participants will be randomized to intervention and control groups equally (allocation ratio, 1:1) using a simple random sampling technique. The randomization assignment will be generated by one independent investigator (PR) using an online random number generation program.

### Concealment mechanism

Sealed opaque envelopes, indicating the allocation of each participant in accordance with the recruitment number, will be prepared in advance. The researcher who will enroll the participants will be given envelopes displaying the allocation at the time of recruitment. Then, a special participant recruitment number will be assigned to each participant for those who meet inclusion and exclusion criteria and give informed consent in chronological order. The sealed envelope containing the allocation for the given recruitment number is opened, and the corresponding participant will be assigned either into the intervention or control group.

### Implementation

Eligible participants will be informed of their randomized assignment once everyone has completed the screening and baseline assessments. The allocation sequence for assigning participants according to the corresponding randomly generated numbers will be produced by a member of the study team (PR) who is not involved in participant recruitment.

## Assignment of intervention: blinding

### Who will be blinded?

The outcome assessors and data analysts will be blinded to the intervention assignment.

### Procedure for unblinding if needed

Participants will not be blinded, given the nature of the study intervention. Single blinding of the outcome assessors and data analysts will be maintained throughout the study.

## Data collection and management

### Plans for assessment and collection of outcomes

The research team will provide sufficient training to the data collectors in order to minimize intra- and inter-observer variations. This includes being familiarized with the research questionnaire and data collection instruments, as well as receiving training on the usage of equipment and measurement techniques. A pre-test will be undertaken using a small group and data will be collected by a well-trained team of data collectors.

Except for DXA scanning and blood investigations, all other tests/measurements will be completed in the study setting. Blood investigations and DXA scans will be undertaken in the Nawaloka Hospitals Laboratory and Radiology department at the Nawaloka Hospitals PLC, Colombo, Sri Lanka. Permission to transport participants to the Nawaloka Hospitals PLC will be obtained from the elderly care institution and National Secretariat for Elders, with participants transported under the supervision of care givers from the elderly care institution.

### Mini nutritional assessment

The MNA short form includes 6 items divided into four areas: anthropometric measurements, global assessments related to lifestyle, dietary questions, and subjective assessment of health and nutrition. The MNA score indicates three different levels of nutritional status: well-nourished (12–14 points), risk for malnutrition (8–11 pints), and under-nourished (< 0–7 points) [24]. MNA will be measured on all residents in both groups, at baseline and after 12 weeks.

### Demographic and medical information

An interviewer-administered semi-structured questionnaire will be used to gather details regarding the participant’s age, and period of stay in the elders’ home. Details related to existing co-morbidities and current medications will be gathered from participants and perusal of their existing medical records.

### Anthropometric measurements

Anthropometric and body composition measurements will be taken by trained data collectors using calibrated equipment according to international recommendations.

- Weight: body weight will be measured using the calibrated scale (Seca 813, Hamburg, Germany), to the nearest 0.1 kg using a digital weighing scale with participants wearing light clothing.
- Height: Height will be taken to the nearest 0.1 cm, as the maximum distance to the uppermost position on the head from heels, with the individual standing barefoot and following full inspiration using a stadiometer (Seca 213 portable stadiometer).
- BMI: Based on the values of weight and height, BMI will be calculated.
- Mid-upper arm circumference (MUAC): The midpoint between the acromion and the olecranon process in the non-dominant arm will be marked when the right elbow is 90° flexed placing the forearm palm down across the trunk. The measurement will be taken using a plastic flexible tape (Seca 201, Germany) to the nearest 0.1 cm when the arm is extended alongside the body with the palm facing upwards.
- Calf circumference (CC): The largest circumference of the calf of the non-dominant leg will be measured using a plastic flexible tape (Seca 201, Germany) to the nearest 0.1 cm in a seated/supine position when the knee and hip are 90° flexed in the two lower limbs.

### Body composition measurements

- Skin fold thickness: Assessed using a Harpenden skin fold caliper (British Indicators, West Sussex, UK) at the triceps skin fold site. All measurements will be taken to the nearest 0.1 mm by the same investigator to maintain consistency and inter-rater reliability. Each skin fold measurement will be taken three times on the non-dominant side and the mean value of the three readings recorded following the standard ISAK protocols. Triceps skinfold measurement will be taken parallel to the long axis of the arm at the triceps skinfold site, the point on the posterior surface of the arm, in the mid-line, at the level of the marked mid-acromial-radial landmark. This point is located by projecting the mid-acromial-radial site perpendicularly to the long axis of the arm around to the back of the arm, and intersecting the projected line with a vertical line in the middle of the arm when viewed from behind. The participant will stand with the arm hanging by the side in the mid-prone position and the elbow extended by the side of the body.
- Dual-energy X-ray absorptiometry (DXA): To estimate fat mass and fat-free mass, DXA measurements will be made with a total body scan (QDR 4500A, fan-beam densitometer, software version 8.21; Hologic, Waltham, USA) that measures the attenuation of X-rays pulsed between 70 and 140 kV synchronously with the line frequency for each pixel of the scanned image. The body scans are to be carried out at an accredited radiography unit.

### Biochemical tests

All biochemical parameters will be tested out at an accredited laboratory (Nawaloka Metropolis laboratory, Nawaloka Hospital PLC) following the standard procedures.

- Full blood count: This will be analyzed using SYSMEX XE-2100 Haematology Automated Analyser.
- HS-CRP: The Roche Cobas c501, which is an in vitro diagnostic test system, will be used to quantitatively determine the C reactive protein (CRP) in human capillary whole blood and serum, EDTA K2/K3 and lithium heparin anticoagulated whole blood and plasma by photometric measurement.
- Serum vitamin D3: The serum will be separated from the 5.0-mL venous blood sample collected and tested within 3 h using MAGMLUMI 2000 analyzer by Competitive Chemiluminescence Immunoassay (CLIA). This test quantitatively measures the sum of both 25-(OH) vitamin D3 (cholecalciferol) and 25-(OH) vitamin D2 (Ergocalciferol) in the specimen.

### Functional capacity

- Hand grip strength: Hand-grip strength (kg) of the dominant or unaffected side of the arm will be measured using a hydraulic handheld dynamometer (SAEHAN). The subject should be tested in the seated position with the elbow flexed at 90°. The handle position should be adjusted to the second joint of the finger just below the handle. The participant will be asked to squeeze the dynamometer at maximal effort for three trials, with a 30-s break between each trial [25]. The maximum strength force of three attempts is recorded for data analysis.
- Activities of daily living (ADL): The ability to perform ADL will be assessed with the Barthel index where a higher score indicates better function. The original index is a three-item ordinal rating scale completed by a therapist or other observer in 2–5 min. Each item is rated in terms of whether the patient can perform the task independently, with some assistance, or is dependent on help based on observation (0 = unable, 1 = needs help, 2 = independent).
- Muscle strength: Peak force of the dominant or unaffected leg’s knee extension (kg) will be measured using a Lafayette Manual Muscle Tester (Model 01163, Lafayette Instrument Company, Lafayette, IN, USA). The instrument will be operated according to the Lafayette manual by trained personnel. The participant should remain seated in a chair with back supported (hip flexed at 90°) with both arms crossed over the chest to prevent him/her from using the hands on the chair and thus enhance the strength applied against the dynamometer. The participant should be instructed to relax all parts of the body and make the effort only in the tested limb. The starting position should involve the knee being tested flexed at 80°. After the examiner’s command to start the test, the participant tries to extend the knee against the resistance applied by the examiner with the dynamometer placed 5 cm above the medial point between the lateral and medial malleolus [25]. The maximum force of three attempts of three trials is recorded for data analysis.

### Frailty

The presence of 3 positive indicators of the five fried model indicators: weight loss, exhaustion, low energy expenditure, slowness, and weakness will be considered as frail.

- Weight loss: Unintentional weight loss of 4.5 kg or more in the past 6 months and/or BMI of less than 18.5 kg/m^2^ at the time of measurement.
- Exhaustion: a little or none of the time responses for the Short Form 12-item Survey (SF-12) question, “how much of the time during the past 4 weeks did you have a lot of energy?”.
- Low energy expenditure: A score < 73 on the Physical Activity Score for the Elderly (PASE) questionnaire.
- Slowness: The average of two readings in the 6m gait speed test and a gait speed of 0.65 ms^−1^ would be considered “slowness.”
- Weakness: Hand grip strength < 25 kg would be considered “weak.”

### Peak expiratory flow rate (PEFR)

The PEFR will be recorded using the mini-Wright’s peak flow meter and the value will be obtained in L/min. Three readings will be taken at a time from each participant and the best among these is taken as the final value [26].

### Blood pressure

The blood pressure of the participants will be measured after 5 min of rest, with the back supported and the legs uncrossed. Constrictive clothes will be removed from the upper arm, and the arm will be rested on a table at the chest level. Blood pressure will generally be recorded to the nearest 2 mmHg using digital blood pressure monitors (Omron Healthcare, Singapore). Three blood pressure values will be collected using standard manometers and the average of those values will be considered the final blood pressure value.

### Dietary assessment

A 24-h dietary recall will be taken to assess the dietary pattern of the residents by a dietitian at baseline, follow-up, and endpoint. Food portion sizes will be obtained using standard household measures such as plates, bowls, cups, glasses, and different spoons. The energy content of each food component will be calculated using standard energy values for the portion sizes. The daily energy intakes are to be calculated using NutriSurvey 2000 software. Furthermore, it will be analyzed for macro and micronutrients using NutriSurvey Software modified with Sri Lankan food composition data.

### Physical assessment

Physical Activity Scale for Elderly: Assessment of physical activity will be done using the Physical Activity Scale for the Elderly (PASE), which is validated as a suitable tool to assess the physical activity of the elderly in community studies [27].

### Cognitive assessment

The Montreal Cognitive Assessment (MoCA) is an internationally accepted, widely used instrument for the assessment of mild cognitive impairment, which has been validated for the Sri Lankan population and it takes about 10–15 min to complete [28]. Cognitive domains such as attention, concentration, memory, executive functions, visuo-constructional skills, calculations, orientation, and conceptual thinking can be assessed separately using MoCA. The MoCA score ranges between 0 and 30 a score of 24 or above will be considered normal and a score below 24 will be categorized as mild cognitive impairment.

#### Plans to promote participant retention and complete follow-up

Investigators will phone the managers and caregivers of the four elderly care institutions to remind participants regarding follow-up before each study visit.

#### Data management

Data will be collected and recorded by a well-trained team of data collectors. Any data collected as part of this research project will be stored in a locked filing cabinet at the Health and Wellness Unit, Faculty of Medicine, University of Colombo, for 5 years, under the supervision of the principal investigator (RJ). The data will be stored in a de-identifiable format. Simultaneously, softcopies will be kept password-protected for the above-prescribed period. Following the completion of the due period, the documents will be shredded and the softcopies will be permanently deleted.

#### Patient public involvement

We aim to ensure that our study is patient-centered and addresses the needs and preferences of the target population. To foster active patient involvement, we have established a patient advisory group (PAG) consisting of caregivers and some other older adults caring for older adults with malnutrition and living in the same elderly care setting. The PAG provided valuable insights regarding the acceptability and feasibility of the oral nutritional supplement intervention. Their involvement included collaboration on participant recruitment strategies, considering their life experiences and recommendations for effective outreach. Further, their contribution involved reviewing and providing feedback on study materials including informed consent forms and questionnaires to ensure they were clear, comprehensive, and patient-friendly. The PAG members will meet regularly throughout the study to maintain ongoing engagement.

#### Confidentiality

The data sheets and electronic data files will not contain any personal information. Each participant will be given a unique study code. The document containing the information on the study code and the identity of the patient will be kept separate from the study data files and data sheets.

#### Plans for collection, laboratory evaluation, and storage of biological specimens for genetic or molecular analysis in this trial/future use

None of the blood samples will be utilized for any other purpose outside the intended tests, and no genetic or molecular analysis will be done. Blood samples will not be given to external bodies and sent abroad. All blood samples will be stored for a month to allow for the completion of the tests and safely destroyed.

## Statistical methods

### Statistical methods for primary and secondary outcomes

Using SPSS version 23 (SPSS Inc., Chicago, IL, USA), parametric and non-parametric statistical tests will be applied for the data analysis. Summary statistics of each group will be computed and presented as mean, standard deviation, and proportion. Using a paired *t*-test, the baseline and end of study characteristics, as well as the laboratory results of the groups, will be compared and a *P* value of <0.05 will be considered significant. Multiple regression analysis will be used to assess other factors influencing weight change. Then non-parametric Mann-Whitney *U* test will be used for asymmetrical continuous variables.

### Interim analysis

The data safety monitoring committee and investigators will consider and decide to terminate the trial, if serious adverse events related to the intervention. Thereafter, an interim analysis will be conducted from the available data. The Sri Lanka Clinical Trial Registry and Ethics Review Committee will have access to interim results.

### Methods for additional analysis (e.g., subgroup analysis)

Subgroup analysis of the primary and secondary outcomes will be performed according to gender.

### Methods in analysis to handle protocol non-adherence and any statistical methods to handle missing data

Each participant’s consumption of the ONS will be monitored on a weekly basis to assess adherence to the intervention. If a participant is unable to consume the supplement continuously for more than five days due to hospitalization or any other reason, they will be considered as “non-adherent” to the intervention. Using Multiple imputation methods accessible in SPSS, the primary outcome data of the participants will be randomized to the intervention but non-adherent participants will be imputed [29].

We expect the proportion of missing data to be below 5% for the primary outcome. We will not perform multiple imputations for the primary outcome unless more than 5% of the data are missing. The secondary outcomes will be assessed in a full analysis set.

### Plans to give access to the full protocol, participant-level data, and statistical code

Full protocol, participant data, and statistical code will be available from the principal investigator upon reasonable request after the publication of the study results.

## Oversight and monitoring

### Composition of the coordinating center and trial steering committee

The coordinating center is located at the Health and Wellness Unit, Faculty of Medicine University of Colombo. The coordinating center consists of research assistants and data collectors. They are responsible for manual operations, facilitation of study recruitment, data collection ensuring the protocol is implemented as planned, reviewing the study progress on a regular basis, and upholding good clinical practice at all times. The primary decision-making body of the study will be the trial steering committee, consisting of the principal investigator and the co-investigators. The steering committee will be responsible for all aspects of the finalization of the study protocol, logistics, and organization of the trial. The information on other committees and groups providing day-to-day support for the trial is summarized in Table 3.

**Table 3:** Committee and supporting groups involved in the trial

**Committee/supporting group**	**Consistency**	**Involvement**
**Recourse persons**	Specialist in sports and exercise medicine	Training the data collectors who will be responsible for obtaining muscle strength parameters, specifically knee extension and hand grip strength.
	Consultant physician in geriatrics	Provide training on various questionnaires used in the study such as the Barthel index, MoCA, IPAQ, PASE, and GAIT assessment.
	Specialist in Nutrition	Provide training on obtaining all anthropometric measurements, screening the participants using MNA-SF, and obtaining 24-h dietary recalls.
**Management of the elders’ home**	President Manager Treasurer	Make necessary arrangements and facilitate the data collection process. This involves coordinating logistics, ensuring a conducive environment for data collection, and managing any administrative tasks related to the trial.
	Matron	Involved in handling the participants with care during the data collection process
**Team of care givers**	Six nurses working in the elderly care home	Actively contribute to the trial by facilitating the supplementation process before bedtime. Nurses will ensure that the participants receive the necessary ONS as per the protocol. Additionally, the team of caregivers is involved in taking video calls for remote monitoring and collection of containers to assess compliance with the intervention. Their involvement strengthens the adherence and compliance of the participants throughout the trial period.

*MoCA* Montreal Cognitive Assessment, *IPAQ* International Physical Activity Questionnaire, *PASE* Physical Activity Scale for the Elderly, *MNA-SF* Mini Nutrition Assessment Short Form, *ONS* oral nutritional supplement

### Composition of the data monitoring committee, its role and reporting structure

An independent board of senior researchers who have no competing interests will be appointed as the data safety monitoring committee. The data safety monitoring committee will review and evaluate the accumulated study data for participants’ safety and study progress on a monthly basis. They will serve in an individual capacity and provide their expertise and recommendations throughout the study and will be responsible for making recommendations to the research team concerning the continuation, modification, or termination of the trial.

### Adverse event reporting and harms

A mechanism will be in place to ensure direct reporting of probable adverse events to the investigator by participants. In case of any adverse events immediate actions, immediate actions will be sought through the administration of the elder’s home.

### Frequency and plans for auditing trial conduct

Representative members from both the trial steering committee and coordinating center will meet monthly to review the trial conduct and evaluate progress. The independent data safety monitoring board and the trial steering committee will meet monthly throughout the trial period in order to notify the Ethics Review Committee of the Sri Lanka Medical Association, if there are any issues. Any protocol amendments will be properly notified to the Ethics Review Committee, and approval will be obtained prior to the implementation.

### Plans for communicating important protocol amendments to relevant parties (e.g., trial participants, ethical committees)

Any amendment to the protocol will be submitted by the principal investigator to be approved by the Ethics Review Committee, Sri Lanka Medical Association.

## Dissemination plans

The findings of the trial will be published in peer-reviewed scientific journals and presented at international scientific conferences.

## Discussion

This study provides a protocol for a RCT to investigate the impact of ONS on anthropometric parameters, body composition, biochemical parameters, functional capacity, frailty, cognitive status, and physical activity level of older adults with malnutrition or at-risk of malnutrition. This may ultimately increase the capacity for independent survival and quality of life of malnourished older adults.

To date, several studies have investigated the effect of ONS on anthropometric parameters, muscle strength, and functional status [17, 18, 25] and used anthropometric measures for the assessment of skeletal muscle mass. For instance, upper arm muscle area was calculated as mid-arm circumference minus π times triceps skinfold thickness [30]. However, such techniques require accurate methodological training and the possibility of propagating measurement errors is high. The major strength of the proposed trial is the use of whole-body DXA scanning to determine changes in body composition [30]. Further, none of the earlier studies focused on the impact of ONS on the bone density of older adults who are malnourished or at risk of malnutrition. DXA will be used to measure bone density and measurements will be compared within and between groups to determine the impact of ONS on the bone density of older adults who are already malnourished or at risk of malnutrition. Another strength of the study is its randomized controlled design. The control group enables the detection of possible effects of the intervention that are beyond an elder’s typical capacity to reverse the status of malnutrition, which is known to be high among older adults [31]. A third strength of the trial is that the intervention is performed in the same elderly care residential setting where it is designed to be implemented.

The study faces several challenges including the high number of tests and the potential increased risk of dropouts. Therefore, participants will be carefully informed about the various tests before and throughout the study to reduce the possibility of dropouts. Further challenges may occur in recruiting participants and their subsequent compliance with supplementation. In order to limit the impact of these challenges, recruitment will be performed by health care personnel, physiotherapists, and nutritionists and caregivers will be thoroughly instructed about the preparation methods of the ONS and advised to monitor carefully the participants’ consumption of the ONS. Additional challenges may occur in transporting participants to the Nawaloka Hospitals Colombo (about 10–20 km away from the elderly care institution) for DXA scanning and blood investigations. Because of the frailty of many participants, special care will be needed during this time.

Finally, a major limitation of the trial may be contamination bias when participants in the control group inadvertently receive the ONS. Participants will be educated as much as possible against contamination and clear information about the purposes of the trial will be provided with supplements provided to caregivers in person. The absence of blinding may also introduce performance bias; however, blinding either participants or investigators is not possible due to the nature of the intervention.

## Trial status

Protocol version 05 (20/03/2023).

Recruitment began in February 2023, and we anticipate recruitment will be completed in 2024.

### Supplementary Information

**Additional file 1.** SPIRIT 2013 Checklist: Recommended items to address in a clinical trial protocol and related documents*.

## Data Availability

The principal investigator RJ and co-investigator PW will have access to the final trial dataset. Any data required to support the protocol can be supplied on request.

## Abbreviations

BMI: Body mass index
BMR: Basal metabolic rate
CC: Calf circumference
CRP: C-reactive protein
DXA: Dual-energy X-ray absorptiometry
EN: Enteral nutrition
ESPEN: European Society for Parenteral and Enteral Nutrition
FBC: Full blood count
IPAQ: International Physical Activity Questionnaire
MNA: Mini Nutrition Assessment
MoCA: Montreal Cognitive Assessment
MUAC: Mid-upper arm circumference
ONS: Oral nutritional supplement
PASE: Physical Activity Scale for the Elderly
PEM: Protein-energy malnutrition
RCT: Randomized control trial
